# A case of Alemtuzumab-induced neutropenia in multiple sclerosis in association with the expansion of large granular lymphocytes

**DOI:** 10.1186/s12883-018-1183-4

**Published:** 2018-10-29

**Authors:** A. G. Vakrakou, D. Tzanetakos, S. Valsami, E. Grigoriou, K. Psarra, J. Tzartos, M. Anagnostouli, E. Andreadou, M. E. Evangelopoulos, G. Koutsis, C. Chrysovitsanou, E. Gialafos, A. Dimitrakopoulos, L. Stefanis, C. Kilidireas

**Affiliations:** 1 1st Department of Neurology, Medical School of Athens, National & Kapodistrian University, Aeginition Hospital, Athens, Greece; 2 Department of Blood Transfusion, Medical School of Athens, National & Kapodistrian University, Aretaieion Hospital, Athens, Greece; 3 Immunology and Histocompatibility Department, Evangelismos Hospital, Athens, Greece

**Keywords:** Multiple sclerosis, Alemtuzumab, Neutropenia, Large granular cells

## Abstract

**Background:**

Alemtuzumab has been demonstrated to reduce the risks of relapse and accumulation of sustained disability in Multiple Sclerosis (MS) patients compared to β-interferon. It acts against CD52, leading primarily to lymphopenia. Recent data have shown that mild neutropenia is observed in 16% of treated MS-patients whereas severe neutropenia occurred in 0.6%.

**Case presentation:**

Herein, we present the case of a 34-year-old woman with relapsing-remitting MS, with a history of treatment with glatiramer acetate and natalizumab, who subsequently received Alemtuzumab (12 mg / 24 h × 5 days). 70-days after the last Alemtuzumab administration, the patient displayed neutropenia (500 neutrophils/μL) with virtual absence of B-cells (0.6% of total lymphocytes), low values of CD4-T-cells (6.6%) and predominance of CD8-T-cells (48%) and NK-cells (47%); while large granular lymphocytes (LGL) predominated in the blood-smear examination. Due to prolonged neutropenia (5-days) the patient was placed on low-dose corticosteroids leading to sustained remission.

**Conclusion:**

This is the first case of a patient with relapsing-remitting MS with neutropenia two months post-Alemtuzumab, with simultaneous presence of LGL cells in the blood and a robust therapeutic response to prednisolone. We recommend testing with a complete blood count every 15 days in the first 3 months after the 1st Alemtuzumab administration and searching for large granular lymphocytes cell expansion on microscopic examination of the peripheral blood if neutropenia develops.

**Electronic supplementary material:**

The online version of this article (10.1186/s12883-018-1183-4) contains supplementary material, which is available to authorized users.

## Background

Alemtuzumab is a humanized monoclonal antibody directed against CD52, a surface glycoprotein with poorly understood role, that mainly is expressed on lymphocytes (B and T cells) and to a lesser magnitude on monocytes, macrophages and eosinophil granulocytes [1]. Mature natural killer (NK) cells, plasma cells, neutrophil granulocytes (neutrophils have approximately 22% the CD52 of lymphocytes), and most importantly, hematological stem cells show little or no expression of CD52 [2]. Alemtuzumab leads to depletion of CD52-positive cells through antibody-dependent cell-mediated cytolysis (ADCC) and complement-dependent cytolysis (CDC) [1, 3]. Recent data from the literature have shown that mild neutropenia is not a rare manifestation in Alemtuzumab-treated MS patients, as approximately 16% of patients developed Grade-I and II neutropenia, yet in unclear time point from drug initiation (Table 1) [4]. Nevertheless, severe neutropenia occurred only in 0.6% of patients (Table 1). Out of these patients, two developed agranulocytosis; one of them was treated with Plasma Exchange (PLEX) and the other with lenograstim [4]. Another study reported that two patients with Grade-III/IV neutropenia were successfully treated with Granulocyte-Colony Stimulating Factor (G-CSF) (Table 1) [5]. Recently, Galgani et al. published a case report of asymptomatic Grade-III neutropenia detected 1 month after first Alemtuzumab course with spontaneous resolution (Table 1) [6]. None of above studies has proposed a mechanism for Alemtuzumab-induced neutropenia. Herein, we present a patient with relapsing-remitting MS with severe neutropenia 2 months post-Alemtuzumab with simultaneous presence of large granular cells in the blood and a robust therapeutic response to prednisolone treatment. We are the first to propose an immunological mechanism for Alemtuzumab-induced neutropenia that merits further investigation in the future.

**Table 1:** Studies showing the incidence and characteristics of neutropenia following alemtuzumab - based therapy in MS patients

Study	Treatment	No of patients	Incidence of neutropenia	Grade of neutropenia	Median time to neutropenia	Median duration of neutropenia	Treatment	Comments
Coles AJ et al., 2012 [16]	1rst year of infusion (24 mg/d)	1/161	0,60%	NA	NA	NA	NA	Febrile neutropenia
Willis et al, 2016 [9]	NA	1/100	1%	NA	Median time to development of acquired autoimmune manifestations was 995 days following first treatment.	NA	NA	None
Gaitán MI et al., 2017 [5]	1rst year of infusion (12 mg/d)	1	case report	IV	4 weeks	3 days	Granulocyte-stimulating factor (300 mg/day for 72 h)	Responsive to 1 cycle of G-SCF, but developed HSV-1 infection that needed advanced antibiotics
Gaitán MI et al., 2017 [5]	1rst year of infusion (12 mg/d)	1	case report	IV-III (two episodes)	6 and 8 weeks (two episodes)	3	Granulocyte-stimulating factor (300 mg/day for 72 h)	Responsive to 2 cycles of G-SCF. Febrile neutropenia andsinusitis that needed iv antibiotics
Baker D et al., 2017 [4]	1rst year of infusion (12 mg/d)	127/811	15,70%	I-II	NA	NA		Data from CARE-MS I and CARE-MS II.
Baker D et al., 2017 [4]	1rst year of infusion (12 mg/d)	5/811	0,60%	III-IV	NA	NA	2 patients developed agranulocytosis,the first teated with PLEX and the other with lenograstim	Data from CARE-MS I and CARE-MS II.
Baker D et al., 2017 [4]	2nd year of infusion	104/808	12,90%	I-II	NA	NA		Data from CARE-MS I and CARE-MS II.
Baker D et al., 2017 [4]	2nd year of infusion	12/808	1,50%	III-IV	NA	NA		Data from CARE-MS I and CARE-MS II.
Vakrakou. et al., 2018 [17]	1rst year of infusion (12 mg/d)	1	case report	III	9 weeks	9 days	Prezolon (25 mg for 3 days and 12,5 mg for another 3 days)	LGL cells predominated in peripheral blood
Galgani S et al., 2018 [6]	1rst year of infusion (12 mg/d)	1	Case report	III/IV	1 month	2 weeks	Resolved spontaneously	

## Case presentation

A 34-year-old female, diagnosed with relapsing-remitting MS since the age of 26, suffered from 2008 to 2013 from recurrent attacks of optic neuritis that partially responded to corticosteroid treatment. The patient was initially treated with glatiramer acetate for 2 years, and then switched to natalizumab (NTM) treatment due to significant clinical relapses. John Cunningham virus seropositivity developed while the patient was receiving NTM intravenously and treatment was discontinued after 24 months. The patient subsequently switched to Alemtuzumab therapy (12 mg/day for 5 days). At the day prior to Alemtuzumab-initiation she had a white blood cell (WBC) count of 14,500/μL (absolute neutrophil count [ANC], 10,900/μL; lymphocytes, 2300/μL) (Additional file 1: Table S1). 9 weeks (Day 65) after the first Alemtuzumab induction therapy, during the standard follow-up, complete blood count revealed severe neutropenia (Grade III) (WBC count, 2000/μL; ANC, 899/μL) (Additional file 1: Table S1), a finding that led to her hospitalization. We tested for the presence of an underlying infection/pathology.

**Fig. 1:**
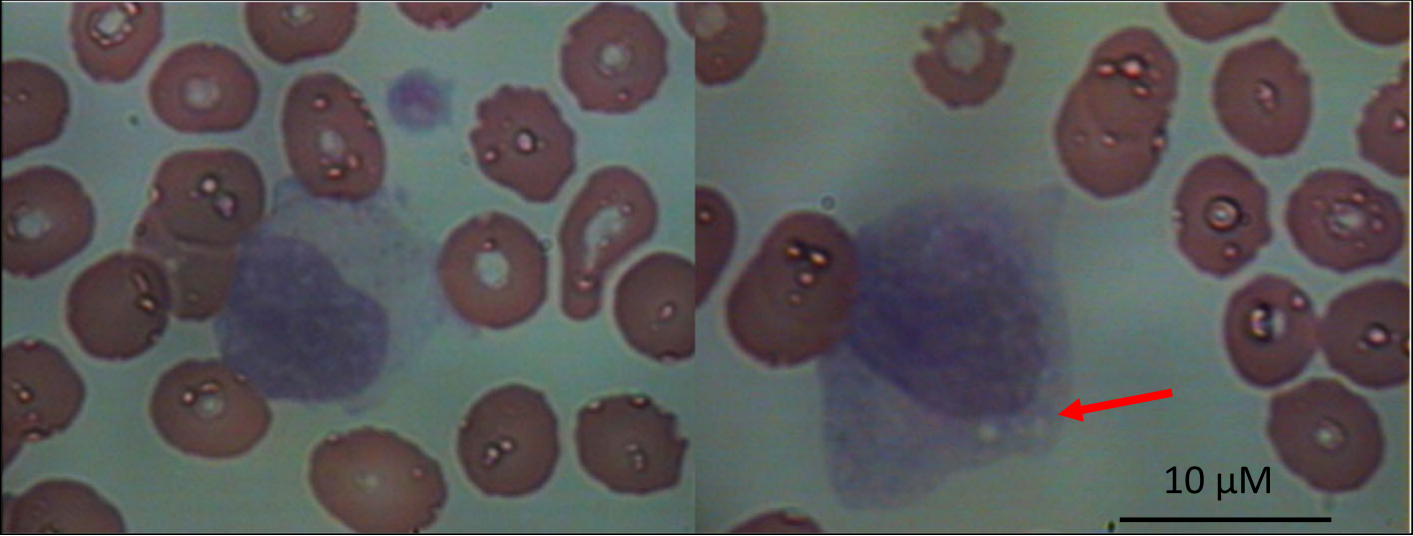
Predominance of large granular lymphocytes in peripheral blood at the onset of Alemtuzumab-induced neutropenia. At the onset of neutropenia, peripheral blood smear analysis (Wright-Giemsa staining) revealed numerous large granular lymphocytes (LGL) (about 80–90%) that had azurophil granules in their cytoplasm (left image) and signs of cystic degeneration (right image, red arrow)

At the onset of neutropenia and throughout its duration, clinical, serological and ultrasonic investigation did not reveal any underlying pathology (Additional file 1: Table S1). At the onset of neutropenia, peripheral blood smear analysis (May-Grünwald-Giemsa staining) revealed numerous large granular cells (LGL cells) (approximately 80–90%) that had variable numbers of randomly distributed azurophilic granules in their cytoplasm (Fig. 1). Neutrophils with apoptotic features were rare. To further verify the nature of LGL cells, immunophenotypic analysis of peripheral blood was performed by flow cytometry. Such analysis showed marked elevation in the percentage of a specific cell-subset that belongs to the NK lineage [CD3-CD(16 + 56+): 47%] (Additional file 1: Table S1). Moreover, the percentage of CD3 + CD8+ T cells was found elevated compared to the baseline levels (before Alemtuzumab initiation). Of notice, the fold increase of CD3 + CD8+ T over baseline values (fold increase: 1.5) was less than that of NK-cells (fold increase: 3.2).

**Fig. 2:**
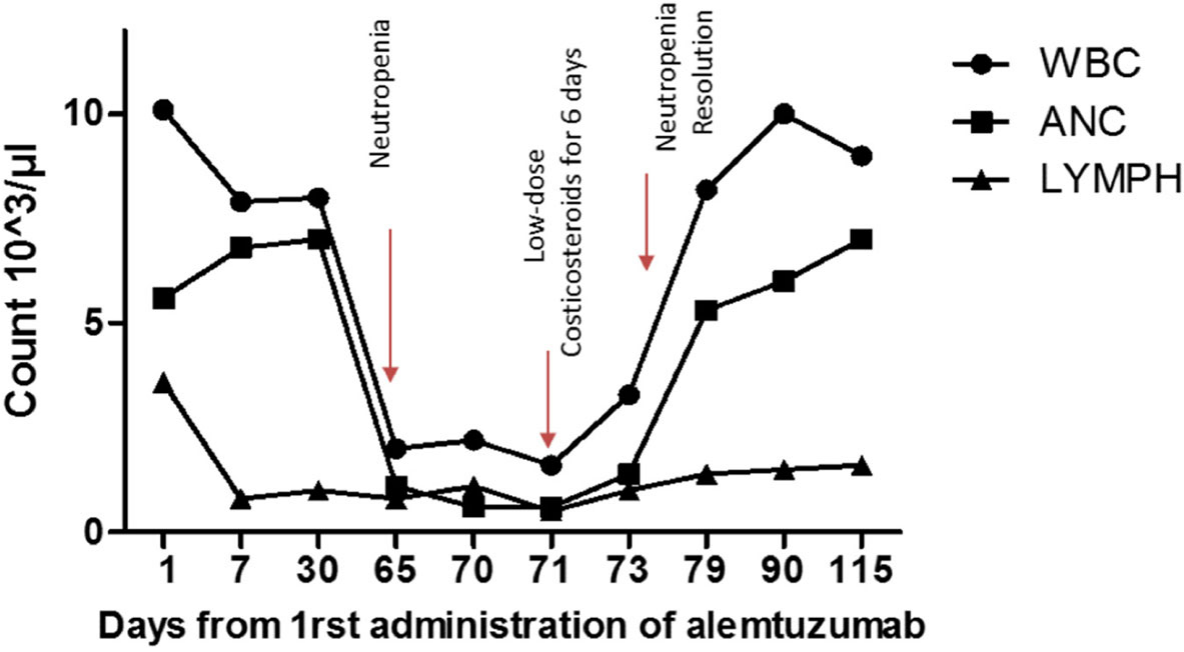
Changes in blood count cells over time. Time points of neutropenia onset, therapeutic intervention and neutropenia resolution are indicated with red arrows. WBC; white blood cells, ANC; absolute neutrophil count, LYMPH; absolute lymphocyte count

At the 70th day post-Alemtuzumab initiation, neutropenia was further exacerbated (ANC = 500 /μL). The occurrence of sustained neutropenia for at least 5 days underscored the need for therapeutic intervention. The patient was placed on corticosteroids (prednisolone 25 mg for 3 days and subsequent dose tapering) and 3 days after, the values of WBC and ANC started to rise, reached normal levels (fourth day) and remained stable for 2 months (Fig. 2). Neutropenia resolution is stable for at least 1 year of follow up. Peripheral blood smear analysis showed that LGL cells were markedly reduced (approximately 50%) after prednisolone initiation and were further diminished 1 month later. Flow cytometry analysis showed that the percentage of NK cells remained increased (48%), whereas the percentage of CD3 + CD8+ showed a significant reduction compared to their levels upon neutropenia development (27.3% versus 48%) (Additional file 1: Table S1). The constellation of neutropenia, along with normal hemoglobin and platelet counts, the expansion in the peripheral blood of LGL cells, in the absence of a common infection, and the responsiveness to corticosteroids were highly suggestive of an ensuing immune-mediated mechanism for Alemtuzumab-induced neutropenia.

During the phase of neutropenia, our patient was in disease remission, with moderate neurologic disability and an EDSS = 2 (pyramidal signs, mild ataxia). We did not perform MRI scanning during the short phase of neutropenia because there was no any disease exacerbation and our patient did not exhibit any new neurological signs. No signs of radiological disease activity were evident during alemtuzumab treatment and as shown in Additional file 2: Figure S1, the lesion size and signal intensity was slightly reduced after 6-months of therapy. Our patient responded well to alemtuzumab, exhibited disease stabilization and was thereof, she was placed in a follow up with neurological examination and assessment of her hematological profile every 1-month, for at least 1 year. Due to prolonged disease remission and the resolution of neutropenia we have not switched to another disease-modifying drug yet.

## Discussion and conclusions

Herein, we present an interesting case of a MS patient who, 2 months following Alemtuzumab treatment, displayed neutropenia with essentially no B cells and very low levels of CD4 T cells; there was a predominance of CD8 T and NK cells, while LGL cells predominated in the blood smear examination. The exact mechanism of early neutropenia associated with Alemtuzumab treatment is a challenging issue. In our case of neutropenia, direct toxic effects of Alemtuzumab on neutrophils are unlikely. Consumption of complement constituents was not noted in the peripheral blood tests. The concept of autoimmune neutropenia, previously described in Alemtuzumab-treated MS patients (very rare), would seem an unlikely causative factor in our case, because such a manifestation would require a longer time interval from Alemtuzumab treatment [7]. Adverse events of Alemtuzumab treatment include infusion-related actions, infections, and secondary autoimmune disorders [8]. Neutropenia may also occur due to secondary autoimmunity after alemtuzumab [9]. The risk of developing secondary autoimmunity is greatest in the first 5 years of follow-up (mean time to development was 995 days following first treatment [9]. So, neutropenia is typically delayed and occurs after immune reconstitution [9]. The most intriguing feature of the present case concerns the expansion of the LGL cell population in the peripheral blood of this Alemtuzumab-treated MS patient. Immunophenotypic analysis showed that LGL cells are likely to be primarily NK and, to a lesser extent, CD8 T cells. Under healthy conditions, LGLs make up 5% to 15% of peripheral blood. LGL are characterized by elevated cytoplasmic:nuclear ratio and plenty azurophilic granules [10]. Initially, LGL were categorized in the lineage of natural killer (NK) cells. Nevertheless, it is now well-known that LGL comprise both cytotoxic T lymphocytes (CTL, CD3+) and NK cells (CD3−), both of which belong to the lymphoid lineage and act as principal mediators of cell-mediated cytotoxicity [10]. Polyclonal expansions of LGL have been observed in healthy elderly and are usually transient, after viral infections such as Epstein–Barr virus and cytomegalovirus, or associated with neoplasms and autoimmune disorders [11].

We suggest that LGL play an active role in the development of neutropenia in our case. Highly suggestive of the operation of an immune-mediated mechanism for the Alemtuzumab-induced neutropenia is the responsiveness to corticosteroids. A short therapeutic protocol with low doses of prezolon led to a constant rise in neutrophil levels and to the normalization of white blood counts. This effect of corticosteroids was accompanied by a reduction in the levels of LGL in the peripheral blood and this effect was stable for at least of 1 month of close monitoring of our patient. Importantly, we performed immunophenotypic analysis of peripheral blood 1 month after the resolution of neutropenia. The percentage of NK cells remained increased, whereas the percentage of CD3 + CD8+ showed a significant reduction compared to their levels upon neutropenia development (Additional file 1: Table S1). This observation suggests that prednisolone treatment did not affect the survival of NK cells, but led to lympholysis of CD3 + CD8+ T cells and favored in overall the reconstitution of neutrophil numbers. This in turn suggests that such CD3 + CD8+ T cells were the main mediators of the neutropenic effect.

Our finding of neutropenia post-Alemtuzumab therapy in a setting of significant cytotoxic T cell-LGL proliferation is highly reminiscent of the potential role of the expanded LGL population in peripheral blood in the pathogenesis of Rituximab-induced neutropenia [12]. Late-onset neutropenia (LON) after rituximab was mainly reported in lymphoma-patients and occurred from 1 month up to 1 year after drug initiation. LGL phenotype has been shown to be associated with neutropenia through various mechanisms such as FAS\FAS ligand mediated neutrophil apoptosis, Fas\Fas ligand independent cytokine\chemokine-related myelosuppression and secretion of inflammatory mediators. Other proposed mechanisms include the presence of antineutrophil antibodies and the role of genetic polymorphisms in the immunoglobulin G (IgG) receptor FCγ RIIIA [13, 14]. An important recent study revealed that 6-months after alemtuzumab treatment CD56bright NK cells were expanded, albeit without alteration in their cytolytic function [15]. Of note, increased numbers of NK cells have also been observed in Hashimoto thyroiditis, which is considered to be a common autoimmune manifestation following alemtuzumab therapy. The exact mechanisms of autoimmunity following alemtuzumab therapy are not fully understood and immune reconstitution changes in cell repertoire could account for immune reactions against self. In this process, the role of CD56bright NK cells as well as cytotoxic CD8 T cells warrants further investigation.

In our patient, neutropenia was observed in a setting of normal hemoglobin level and platelet count (Additional file 1: Table S1), thus making the possibility of toxic (chemotherapy-related) or other (e.g. viral) underlying factors being responsible for our observations very remote. To our knowledge this if the first study to show the association of Alemtuzumab with LGL proliferation and neutropenia development. The exact triggering mechanism of LGL expansion is elusive until now. The present case indicates that clinicians should be aware of this particular side effect of Alemtuzumab, strongly implying the need of a close laboratory evaluation of patients after drug administration. Taken together, the results reported here challenge the currently published notion that severe Alemtuzumab-related neutropenia in the setting of MS should be treated with G-SCF. Considering the published data of the detrimental effect of G-SCF in MS disease evolution, we suggest prednisolone as an alternative therapeutic option. Therefore, we suggest complete blood count analysis every 15 days during the first 3 months following drug initiation and search for LGL cell expansion if neutropenia evolves. Moreover, we suggest early therapeutic intervention for Alemtuzumab-induced Grade-III neutropenia with low-dose corticosteroids.

## Additional files

Additional file 1: **Table S1.** Table describing the hematological and serological profile of our patient with Alemtuzumab-induced neutropenia. Whole blood analysis, immunophenotypic analysis and serological analysis of parameters before, at the onset of neutropenia, throughout its duration and after neutropenia resolution. (DOCX 17 kb)

Additional file 2: **Figure S1.** Brain MRI scanning for our patient before and after Alemtuzumab initiation. Brain MRI scanning revealed decline in lesion size and signal intensity 6-months after alemtuzumab initiation compared to baseline (2 months prior to alemtuzumab initiation). (DOCX 9019 kb)

## Abbreviations

ADCC: Antibody-dependent cell-mediated cytolysis
ANC: Absolute neutrophil count
CDC: Complement-dependent cytolysis
CTL: Cytotoxic T lymphocytes
G-CSF: Granulocyte-Colony Stimulating Factor
LGL: Large granular lymphocytes
LON: Late onset neutropenia
MS: Multiple Sclerosis
NK: Natural killer cells
NTZ: Natalizumab
PLEX: Plasma exchange
RRMS: Relapsing remitting multiple sclerosis
WBC: White blood cells
