# Supplementary material for: A case of Alemtuzumab-induced neutropenia in multiple sclerosis in association with the expansion of large granular lymphocytes
Source: BMC Neurol. 2018 Oct 29;18:178. doi: 10.1186/s12883-018-1183-4 (PMC6206708; doi:10.1186/s12883-018-1183-4)
Supplement: Supplementary file 1 — Table S1. Table describing the hematological and serological profile of our patient with Alemtuzumab-induced neutropenia. Whole blood analysis, immunophenotypic analysis and serological analysis of parameters before, at the onset of neutropenia, throughout its duration and after neutropenia resolution. (DOCX 17 kb) [file 12883_2018_1183_MOESM1_ESM.docx]

| Tests (normal range) | Day 1 (prior to alemtuzumab) | Days after 1rst alemtuzumab administration | | |
| --- | --- | --- | --- | --- |
|  |  | Day 7 | **Day 71** | Day 115 |
| **WHOLE BLOOD ANALYSIS** | | | | |
| WBC x 10^3/μL (4-9) | 10,1 | 7,90 | **1,6** | 9 |
| NEUTROPHILS 10^9/L (1,5-6,6) | 5,6 | 6,8 | **0,6** | 7 |
| LYMPHOCYTES 10^9/L (1,5-3,5) | 3,6 | 0,8 | **0,5** | 1,6 |
| PLATELETS 10^3/μl (150-400) | 315 | 221 | 336 | 400 |
| HGB g/dL (12-16) | 14,0 | 15,6 | 14,4 | 13,0 |
| HCT % (37-47) | 42,2 | 46,4 | 42,7 | 39,7 |
| **IMMUNOPHENOTYPE ANALYSIS OF WHOLE BLOOD** | | | | |
| CD3+ (69,5-81,3%) | 70,6 | ND | 52 | 37 |
| CD3+ CD4+ (40-55%) | 38,7 | ND | 6.6 | 10,3 |
| CD3+CD8+ (22-30%) | 31,4 | ND | **48,5** | 27,3 |
| CD19+ (7,3-17,6%) | 14 | ND | 0,6 | 13 |
| CD3-CD(16+56)+ (5,9-15,1%) | 14,6 | ND | **47,2** | **48** |
| **SEROLOGICAL PARAMETERS** | | | | |
| ANA | Negative | ND | Negative | ND |
| RF mg/dl (0-15) | <10,60 | ND | <10,60 | ND |
| C3 mg/dl (90-180) | 120,00 | ND | 140,00 | ND |
| C4 mg/dl (10-40) | 35,50 | ND | 35,50 | ND |
| IgG mg/dl (700-1600) | 800 | ND | 792,00 | ND |
| IgA mg/dl (70-400) | 160,00 | ND | 182,00 | ND |
| IgM mg/dl (40-230) | 95,60 | ND | 95,60 | ND |
| CMV, HSV1+2, VZV, EBV, Parvo B19, Adenovirus, Measles virus IgM | ND | ND | Νegative | ND |

**Additional file 1: Table S1**
