# Supplementary material for: A case of Alemtuzumab-induced neutropenia in multiple sclerosis in association with the expansion of large granular lymphocytes
Source: BMC Neurol. 2018 Oct 29;18:178. doi: 10.1186/s12883-018-1183-4 (PMC6206708; doi:10.1186/s12883-018-1183-4)
Supplement: Supplementary file 2 — Figure S1. Brain MRI scanning for our patient before and after Alemtuzumab initiation. Brain MRI scanning revealed decline in lesion size and signal intensity 6-months after alemtuzumab initiation compared to baseline (2 months prior to alemtuzumab initiation). (DOCX 9019 kb) [file 12883_2018_1183_MOESM2_ESM.docx]

**Additional file 2: Figure S1**

**Additional file 2: Figure S1 Legend:** Brain MRI scanning revealed decline in lesion size and signal intensity (FLAIR and T2 images) 6-months after alemtuzumab initiation (lower panel) compared to baseline (2 months prior to alemtuzumab initiation, upper panel). Lesions at cerebral hemispheres as well as periventricular lesions and lesions in pons exhibit no gadolinium (GD) enhancement (T1 + GD images), at both time points.
